# LXG Toxins of Bacillus Velezensis Mediate Contact-Dependent Inhibition in a T7SS-Dependent Manner to Enhance Rhizosphere Adaptability

**DOI:** 10.3390/ijms26062592

**Published:** 2025-03-13

**Authors:** Xia Shu, Xiting Sun, Kesu Wang, Yan Duan, Yunpeng Liu, Ruifu Zhang

**Affiliations:** 1 State Key Laboratory of Efficient Utilization of Arid and Semi-Arid Arable Land in Northern China, The Institute of Agricultural Resources and Regional Planning, Chinese Academy of Agricultural Sciences, Beijing 100081, China; 2 College of Life Science and Technology, Huazhong Agricultural University, Wuhan 430070, China; 3 Jiangsu Provincial Key Lab for Organic Solid Waste Utilization, National Engineering Research Center for Organic-Based Fertilizers, Jiangsu Collaborative Innovation Center for Solid Organic Waste Resource Utilization, Nanjing Agricultural University, Nanjing 210095, China

**Keywords:** *Bacillus velezensis*, T7SS, LXG toxins, contact-dependent inhibition, rhizosphere competition, biofilm

## Abstract

Rhizosphere bacteria always compete intensely for ecological niches, employing various strategies to inhibit the growth of microbial competitors. One such strategy, contact-dependent inhibition (CDI), involves the direct delivery of toxic proteins into competing neighboring bacteria by a secretion system, leading to the inhibition of their growth. However, the ecological function of CDI competition in the natural environment remains unclear. In this study, we examined the role of the type VII secretion system (T7SS) substrate LXG domain-containing protein in the rhizobacterium *Bacillus velezensis* SQR9 and found that SQR9 encodes LXG toxins mediate contact-dependent inhibition against other *Bacillus* strains in biofilms. Transcriptional analysis revealed that the expression of these lxg genes is induced by root exudates and positively correlates with that of the T7SS gene cluster. We further confirmed that the survival of the mutants deficient of the LXG toxins was significantly decreased in natural soil. These findings highlight the critical role of T7SS and its substrate LXG toxins in competition of *Bacillus* species in the rhizosphere, providing new insights into the ecological importance of CDI in natural environments.

## 1. Introduction

The rhizosphere is a complex and competitive environment [1]. Bacteria inhabiting the rhizosphere often occupy overlapping ecological spaces and form biofilms around the host [2,3,4]. To establish and maintain their ecological niches, bacteria compete for essential nutrients and engage in antagonistic interactions with other microbial competitors [5,6,7]. A common strategy employed by bacteria involves the secretion of diffusional toxins [8,9]. These toxins are typically released into the surrounding environment, where they indiscriminately kill target bacteria [10]. However, as most rhizobacteria live in biofilms, the extracellular matrix provides a degree of resistance to antimicrobial compounds, thus, reducing the efficacy of such a strategy [11,12,13]. To effectively compete with neighboring bacteria, they have evolved contact-dependent inhibition (CDI) that relies on the secretion system, which directly delivers protein toxins into competing bacteria cells, thereby inhibiting their growth or killing them [14,15,16,17,18,19]. CDI was initially identified in Gram-negative bacteria and has been shown to possess widespread and cross-species effects [20,21,22]. To date, several secretion systems have been implicated in CDI, such as T1SS, T4SS, and T6SS [21]. The CDI effector proteins exhibit a diverse range of types and functions, but all of them target the essential processes for bacterial survival, such as cell wall synthesis, cell membrane permeability and potential, nucleic acids, and critical metabolic pathways involved in growth and cell division [21].

Recently, a secretion system unique to Gram-positive bacteria, named as a type VII secretion system (T7SS), has been reported to mediate the competition of bacteria through a CDI mechanism. This process depends on protein containing an LXG (Leu-x-Gly, x means to any amino acid) motif, with the C-terminal domain encoding polymorphic toxins [23,24,25]. Initially, the LXG toxin protein mediating intraspecies competition was found in *Staphylococcus aureus*, where it was reported that there is an LXG domain-containing protein that encodes a nuclease in C-terminal, targeting and killing mutant cells without antitoxin protein via an Esx secretion pathway [26]. Due to the differences in outer cellular structure between Gram-positive and Gram-negative bacteria, secretion systems such as the T4SS and T6SS in Gram-negative bacteria often employ needle-like complexes to directly inject toxins into adjacent cells [15,16,27,28,29,30,31,32,33,34]. In contrast, the T7SS in Gram-positive bacteria typically secretes effector proteins to the extracellular environment, and the mechanisms by which these extracellular effectors recognize and enter target cells remains a mystery. Research on *Bacillus* T7SS-mediated CDI competition among bacteria has only been reported in recent years, with reports that *Bacillus subtilis* NCIB3610 encodes a range of LXG toxins and mediates CDI in biofilms, with the crucial involvement of the T7SS ATPase YukC and the WXG100 family substrate YukE [35]. However, the regulatory mechanism and ecological significance of T7SS-mediated CDI in *Bacillus* remains poorly understood.

Our previous study has shown that T7SS mediates rhizosphere probiotics and plant root interactions, enhancing the rhizosphere colonization ability of *Bacillus* [36]. Since T7SS also highly improved the rhizosphere colonization of *Bacillus* in natural soil, which is rich for bacteria, we hypothesize that the T7SS in *Bacillus* species may plays a role in competition within rhizosphere. *Bacillus velezensis* SQR9 is a well-studied plant growth-promoting rhizobacteria with capabilities in enhancing plant growth and controlling plant diseases, which have already been applied in agricultural production. In this study, we demonstrate that the beneficial rhizobacterium *Bacillus velezensis* SQR9 encodes seven pairs of LXG toxin-antitoxin proteins and participates in CDI-mediated interspecies competition in a T7SS and YukE-dependent manner and propose that YukE facilitates cell–cell adhesion, thus, contributing to CDI. Transcriptional analysis revealed that the expression of *lxg* genes is induced by root exudates. Finally, we show that *Bacillus velezensis* SQR9 secretes YukE along with LXG toxins via T7SS, enhancing its competitiveness in the rhizosphere.

## 2. Results

### 2.1. B. velezensis SQR9 Encodes LXG Domain-Containing Toxins to Inhibit Other Bacillus Strains

**Figure 1:**
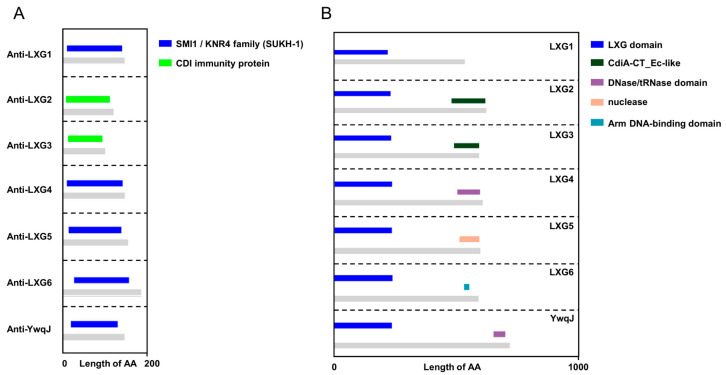
*Bacillus velezensis* SQR9 encodes seven pairs of Toxin-Antitoxins of LXG. Protein sequence alignment and functional domain prediction were using the SMART (https://smart.embl.de) and InterPro (https://www.ebi.ac.uk/interpro) databases. The figure illustrates the annotation results of the target protein obtained from both SMART and InterPro. (**A**) Structural domains of the immune proteins. Gray indicates the whole protein; colors indicate the immune functional domain of CDI toxins. (**B**) Structural domains of the LXG toxin proteins. Gray indicates the whole protein, blue indicates the conserved N-terminal LXG domains, and colors indicate different C-terminal toxin functional domains.

The LXG domain, characterized by the Leu-x-Gly motif, is a distinctive feature recognized by the Type VII Secretion System (T7SS). Proteins harboring this motif at their N-terminus are potential T7SS substrates, with their C-termini typically encoding polymorphic toxin domains that facilitate CDI competition between bacteria. To prevent self-toxicity, antitoxin proteins are usually encoded alongside toxin genes in the genome, sharing a common promoter. In *Bacillus velezensis* SQR9, we identified seven toxin genes encoding proteins with the LXG domain: *lxg1* (V529_04770), *lxg2* (V529_05730), *lxg3* (V529_11460), *lxg4* (V529_18210), *lxg5* (V529_19080), *lxg6* (V529_21700), and *ywqJ* (V529_36050). Structural domain analysis revealed that these proteins encode nucleases in their C-terminal regions (Figure 1B). Phylogenetic analysis indicated that LXG1 and LXG2, LXG3 and LXG4, and LXG5 and LXG6 are closely related, respectively, while YwqJ forms a distinct branch (Figure S3).

**Figure 2:**
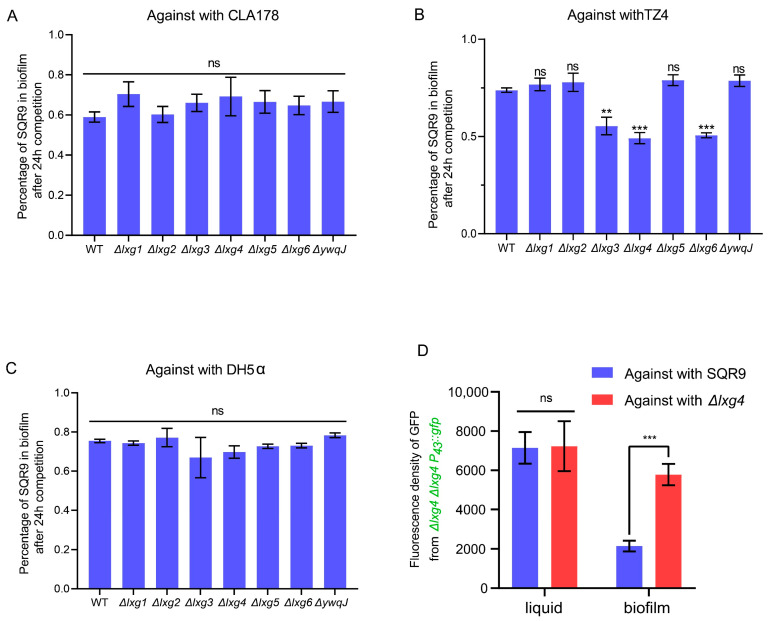
*Bacillus velezensis* SQR9 LXG toxins mediate interspecies competition within biofilms. (**A**–**C**) Interspecies competition experiments were performed on solid LB medium, SQR9 WT, and the derived mutants as the competitor strain, *Bacillus velezensis* CLA178, *Bacillus proteolytics* TZ4, and *E.coli* DH5α as the recipient strain, equally mixed in pair with competitor and recipient cells, respectively, competing within biofilm for 24 h at 30 °C. qPCR was used to measure the copies of the competitor and data were calibrated with the DNA concentration of the biofilm. (**D**) The lacking antitoxin for LXG4 toxin mutant Δlxg4Δantilxg4 was introduced, P43::gfp encoding gene is for distinguished, and the fluorescence level represents the survival situation. SQR9 WT and the lacking LXG4 toxin mutant Δlxg4 is the competitor strain, Δlxg4Δantilxg4 P43::gfp is the recipient strain, equally mixed in pairs with the competitor and recipient cells, respectively, competing within biofilm or liquid LB medium for 24 h. Fluorescence levels of the GFP-expressing were detected and represented for recipient cells survival rate. All experimental data are represented as mean ± SEM (*n* = 3). The significant difference was analyzed by unpaired two-tailed Student’s *t*-test, “ns” indicates *p* ≥ 0.05, “**” indicates *p* < 0.01, and “***” indicates *p* < 0.001.

To identify the corresponding antitoxin proteins for these seven LXG toxins, we noted that bacterial immune proteins typically contain SmI1/Knr4 (Pfam: PF09346) domains. Sequence alignment identified seven genes adjacent to the lxg genes, each encoding immune protein domains that match the characteristic of SmI1/Knr4 or CDI immune protein, which we named as *antilxg1* (V529_04750), *antilxg2* (V529_05720), *antilxg3* (V529_11430), *antilxg4* (V529_18220), *antilxg5* (V529_19070), *antilxg6* (V529_21690), and *antiywqJ* (V529_36040) (Figure 1A). To confirm whether these antitoxin proteins neutralize LXG toxicity, we selected lxg4 as the target gene and constructed knockout mutants for the corresponding antitoxin gene. We found that deleting the antitoxin gene alone was lethal to the bacteria. Therefore, we generated a mutant Δlxg4Δantilxg4, which lacked both the *antilxg4* and *lxg4*. As expected, Δlxg4Δantilxg4 was unable to withstand the toxicity of the LXG4 toxin protein secreted by SQR9 (Figure 2D).

To investigate whether the seven LXG proteins encoded by SQR9 participate in bacterial competition, we constructed seven mutants, each lacking one of the lxg encoding genes, and confirmed that these deletions did not affect the growth or biofilm formation (Figures S1 and S2). We then equally mixed and cultured these seven lxg deletion mutants with other bacterial strains, respectively, allowing them to compete within a biofilm for 24 h, to investigate whether the loss of a single LXG toxin protein weakens the competitive advantage of SQR9. The results showed that when competed with the same species *B. velezensis* CLA178 or the typical Gram-negative bacterium *E. coli* DH5α, the LXG proteins did not confer any competitive advantage to the toxin-encoding strain (Figure 2A,C). However, when competing with the different species *Bacillus proteolyticus* TZ4, the deletion of *lxg3*, *lxg4*, or *lxg6* caused a significant reduction in the survival rate of SQR9 (Figure 2B). We further examined the LXG4 toxic effect in liquid and biofilm environments and found that this competitive effect was observed only in biofilm environments (Figure 2D). These results indicate that the LXG toxin secreted from SQR9 showed typical CDI characteristics. To further investigate whether the LXG toxin could killed the target cells totally, we increased the initial inoculation rate of the toxin-secreting competitor strain to 20-fold in the competition experiment and observed that the recipient strain, lacking the antitoxin gene *antilxg4*, was not completely killed by the LXG4 toxin (Figure S4). Next, we replaced the *lxg4* gene promoter with the constitutive P43 promoter and generated a competitor strain that overexpresses the LXG4 toxin, named SQR9 P43::*lxg4*. When SQR9 P43::*lxg4* competed with the recipient cells, the results showed that even after increasing the initial inoculation of SQR9 P43::lxg4 20-fold, the recipient strain whose antilxg4 gene was deleted still was not completely dead and maintained a stable survival rate of approximately 10% (Figure S4). These results suggest that the LXG toxin proteins encoded by *B. velezensis* SQR9 are involved in interspecies competition within biofilms and mediate contact-dependent inhibition, rather than killing the target cells.

### 2.2. The Toxic Effects of the LXG Toxins Were Dependent on T7SS

**Figure 3:**
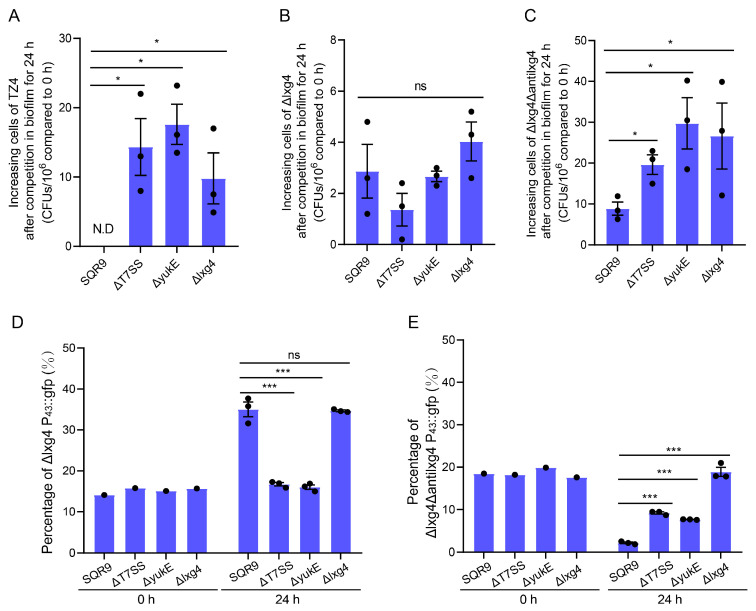
*Bacillus velezensis* SQR9 LXG toxin effects are dependent on T7SS. Interspecies competition experiments performed on solid LB medium, the competitor strain and recipient strain were equally mixed in pairs, respectively, competing within biofilm for 24 h. (**A**–**C**) The recipient strains were marked by chloramphenicol resistance cassette for survival detection before the competition. SQR9 WT, the T7SS-related mutants ΔT7SS, ΔyukE, and the LXG4 toxin deficient mutant were treated as the competitor strain; TZ4, Δlxg4, and Δlxg4Δantilxg4 were treated as the recipient strain, LB solid medium containing 10 mg/L of chloramphenicol was used to measure the survival recipient cells after competition, data were calibrated with the initial inoculation cells. (**D**,**E**) The recipient strains were marked by the P43::gfp encoding gene in the genome for recognition before the competition. Flow cytometric analysis of the GFP-expressing rate to represent the recipient strain survival ratio. (**D**) SQR9 WT, the T7SS-related mutants ΔT7SS, ΔyukE, and the LXG4 toxin deficient mutant were treated as the competitor strain; Δlxg4 P43::gfp were treated as the recipient strain. (**E**) SQR9 WT, the T7SS-related mutants ΔT7SS, ΔyukE, and the LXG4 toxin deficient mutant were treated as the competitor strain; Δlxg4Δantilxg4 P43::gfp were treated as the recipient strain. All experimental data are represented as mean ± SEM (*n* = 3). The significant difference was analyzed by unpaired two-tailed Student’s *t*-test, “N.D” indicates not dectected, “ns” indicates *p* ≥ 0.05, “*” indicates *p* < 0.05, “***” indicates *p* < 0.001.

The LXG toxin effects of *Bacillus subtilis* NCIB3610 have been demonstrated to depend on the T7SS ATPase protein YukC and the WXG100 family substrate YukE [35]. To determine whether the LXG toxins in *Bacillus velezensis* SQR9 also rely on T7SS, we labeled the recipient strains by introducing a chloramphenicol resistance cassette for accurate identification of them in competition experiments. We selected ΔT7SS (lacking the T7SS secretory machine-related gene), ΔyukE (lacking the *yukE* gene), and Δlxg4 (lacking LXG4 toxin) as competitor strains, mixed equally in pairs with the recipient strains TZ4, Δlxg4, and Δlxg4Δantilxg4, respectively, allowing them to compete within biofilm for 24 h, and the survival of the recipient strains was detected. As expected, both ΔT7SS and ΔyukE showed a significant reduction in their ability to inhibit the growth of TZ4 and the antitoxin-deficient mutant Δlxg4Δantilxg4 compared with that of the wild-type (Figure 3A,C). However, T7SS-related mutant strains showed no difference in inhibiting the mutant Δlxg4, which contained the *antilxg4,* compared with wild-type SQR9, which did (Figure 3B). We then used the Δlxg4 P43::gfp and Δlxg4Δantilxg4 P43::gfp as recipient strains and mixed them in pairs with competitor strains SQR9, ΔT7SS, ΔyukE, and Δlxg4, respectively, for competition. After 24 h of competition, we measured the proportion of GFP expression cells in each mixed biofilm by flow cytometry, assessing the survival of recipient bacteria. As expected, the growth of the antitoxin lacking mutant Δlxg4Δantilxg4 P43::gfp was not inhibited by the Δlxg4, neither by the strains lacking T7SS-related elements or the *yukE* gene (Figure 3E). This suggests that the LXG toxin effect is dependent on both T7SS and YukE. 

Since Δlxg4 P43::gfp still retained the antitoxin of LXG4, we observed that none of SQR9, ΔT7SS, ΔyukE, and Δlxg4 reduced the proportion of Δlxg4 P43::gfp in a co-culture experiment detected by counting CFUs (Figure 3B). However, in a same experiment setup we measured the proportion of the Δlxg4 P43::gfp using flow cytometry for detecting the fluorescent cells when co-cultured with SQR9, ΔT7SS, ΔyukE, and Δlxg4, respectively. Here, we observed that the proportion of Δlxg4 P43::gfp were highly increased after coculture for 24h with SQR9 and Δlxg4, and not ΔT7SS or ΔyukE, which is inconsistent with the results obtained by CFUs counting of the receipt Δlxg4 on Petri dishes (Figure 3B,D). Given the limitation of flow cytometry in detecting cells that adhere together, we hypothesize that the observed increase in fluorescent “cells” may result from cell–cell adhesion between SQR9/Δlxg4 P43::gfp and Δlxg4/Δlxg4 P43::gfp, but not between DT7SS/P43::gfp or ΔyukE/Δlxg4 P43::gfp in the co-culture system. This aggregation phenomenon could lead to an apparent reduction in fluorescent cell counts during flow cytometric analysis, as clustered cells may be enumerated as single events. Our findings suggest that YukE may potentially mediate intercellular adhesion; even further experiment is need to confirm this hypothesis. 

### 2.3. lxg Genes Expressions Were Induced by Plant Root Exudates

**Figure 4:**
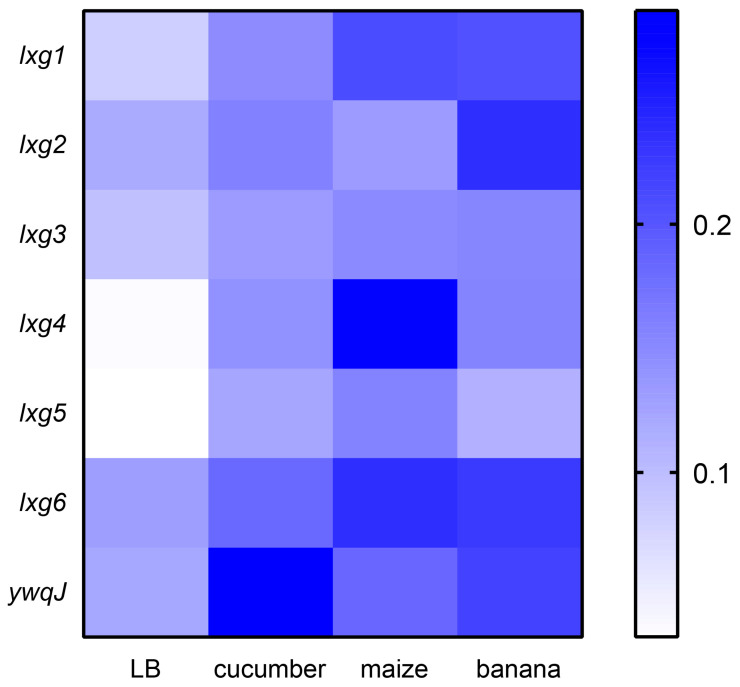
*Bacillus velezensis* SQR9 lxg genes are upregulated by root exudates. Heatmap of transcriptional levels of *lxg* genes in response to different plant root exudates from RNA-seq dataset. The FPKM were normalized by sum, colors represent the expression levels: blue indicates high expression and white indicates low expression, *n* = 3.

*Bacillus velezensis* SQR9 is a plant growth-promoting bacteria inhabiting the rhizosphere, which is highly influenced by root exudates. The root exudates support a diverse and dense microbial community, making the rhizosphere a hotspot for microbial competition. We wondered whether SQR9 could sense root exudates to decide the synthesis of LXG toxin for competition or not. To verify this hypothesis, we analyzed the RNA-seq data from SQR9 cultured in medium with root exudates from different plants [37]. As expected, the expressions of lxg genes in SQR9 were significantly induced upon all kinds of root exudates treatment, with lxg4 and lxg5 showing the greatest expression induction by the root exudates. Furthermore, different lxg genes responded variably to different root exudates (Figure 4). Since root exudates from different plants often recruit and shape different bacterial communities [38], the differential response of lxg genes to diverse root exudates may reflect that the diverse LXG toxin proteins are used to competing with different bacterial species by SQR9. These results suggest that LXG toxins are likely involved in rhizosphere microbial competition, thus, promoting the adaptability of *B. velezensis* SQR9 in the rhizosphere.

### 2.4. lxg Is Positively Correlated with T7SS and yukE Expression

**Figure 5:**
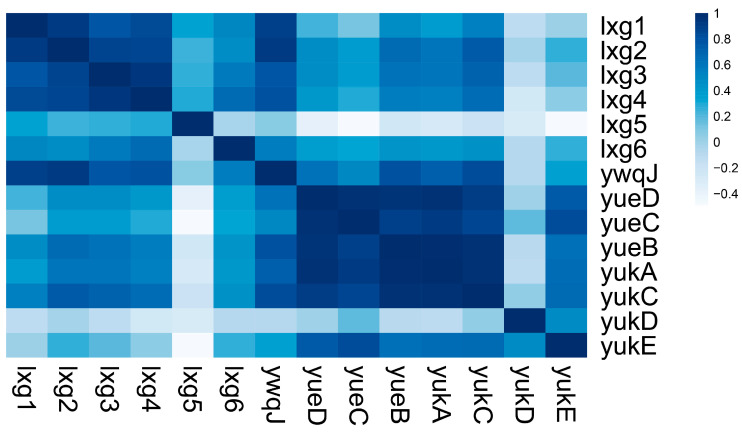
*Bacillus velezensis* SQR9 lxg and T7SS gene cluster transcription are positively correlation. Heatmap of the transcriptional levels correlation between T7SS gene cluster and lxg genes. Pearson correlation was calculated by R, colors indicate transcriptional correlation (R value): blue indicates high correlation and white indicates low correlation. Data were collected from a dataset, containing 72 independent RNA-seq samples, and normalized by sum and transformed to relative log expression values. The R-value and *q*-value of the heatmap is provided in Supplementary Table S3.

In our previous studies, we demonstrated that the T7SS of *Bacillus velezensis* SQR9 mediates the secretion of the WXG100 family protein YukE, which interacts with plant roots and promotes root colonization [36]. LXG toxins have been implicated in interspecies competition, a process that also depends on YukE. To further explore the relationship between LXG toxins, T7SS, and YukE, we conducted a Pearson correlation analysis of the transcriptional FPKM values between the seven lxg genes and the T7SS gene cluster across 72 RNA-seq datasets [37,39,40,41]. Our analysis revealed that the transcription of *lxg1*, *lxg2*, *lxg3*, and *lxg4* showed a significant positive correlation. Furthermore, except for *lxg5*, the transcription of the remaining six lxg genes showed positive correlation with the T7SS gene cluster (Figure 5). These findings suggest that the LXG toxins and WXG100 substrates secreted by the T7SS are likely to work synergistically to enhance the adaptability of SQR9 in the rhizosphere. 

### 2.5. LXG Toxin Enhances B. velezensis SQR9 Ability to Compete with Rhizosphere Soil Bacteria

**Figure 6:**
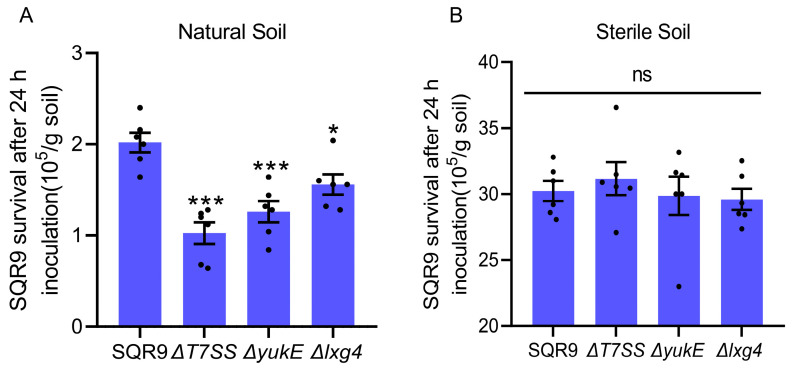
*Bacillus velezensis* SQR9 LXG toxin enhancing rhizosphere soil competiveness via T7SS. SQR9 WT, the T7SS-related mutants ΔT7SS, ΔyukE, and the LXG4 toxin deficient mutant were inoculated into soil to a final concentration of 10^7^ cells/g soil. The survival cells of each strain were calculated at 24 h post-inoculation, and the data were normalized by soil weight. (**A**) Strains were inoculated into the natural rhizosphere soil. (**B**) Strains were inoculated into the sterile rhizosphere soil. All experimental data are represented as mean ± SEM (*n* = 6). The significant difference was analyzed by unpaired two-tailed Student’s *t*-test, “ns” indicates *p* ≥ 0.05, “*” indicates *p* < 0.05, “***” indicates *p* < 0.001.

To further explore the ecological function of the CDI effect of the LXG toxin, we inoculated wild-type SQR9, ΔT7SS, ΔyukE, and Δlxg4 in rhizosphere soil, respectively, and compared their survival after 24 h. The results showed that the survival of each strain in sterile rhizosphere soil was significantly higher than that in natural rhizosphere soil, and there was no difference among all strains (Figure 6B). In contrast, compared to WT SQR9, the mutants ΔT7SS, ΔyukE, and Δlxg4, who lost LXG4 toxin effects in CDI process, showed a significant decrease in survival in natural soil (Figure 6A). These results demonstrated that SQR9 secreted LXG toxin via a T7SS-dependent way and cooperated with YukE to compete with the native bacteria, thereby enhancing the adaptability of SQR9 in the rhizosphere. These findings support the hypothesis that LXG toxins and T7SS play a role in bacterial competition in the rhizosphere.

## 3. Discussion

The rhizosphere is a hotspot for bacterial competition. Bacteria inhabiting the rhizosphere need to interact efficiently with their host to gain competitive advantages against other microbial competitors to take superior ecological niches. Biofilm plays a crucial role in the beneficial interactions between bacteria and plants at the root surface. Our previous studies demonstrated that the T7SS enhances the rhizosphere colonization ability of rhizobacterium *Bacillus velezensis* SQR9 [36]. In this study, we found that SQR9 encodes LXG toxins, mediating interspecies competition through CDI in a T7SS-dependent manner (Figure 2 and Figure 3). Moreover, the genes encoding these LXG toxins are significantly induced by root exudates (Figure 4). This study confirms that T7SS enhanced the competition of SQR9 with other bacteria through LXG toxins in rhizosphere soil (Figure 6). 

It is noteworthy that we observed that the LXG toxin secreted by SQR9 only mediates inhibition without completely eliminating competing bacteria (Figure S4), which brings us to a study in which Michael J. Bottery et al. demonstrated that the CDI system of *Escherichia coli* significantly reduces the ability of susceptible target bacteria to expand their survival range during growth and markedly alters the bacterial community and spatial organization in biofilm [42]. Given that the antibacterial effect of CDI entirely relies on close contact between cells, the remnant cells of susceptible bacterial after competition may evade such close contact with the toxin-secreting cells by altering their spatial position within the biofilm, thereby maintaining survival, but resulting in a survival disadvantage due to the prevention of the further expansion of their territory.

The specific mechanism of LXG toxin effects is still not well studied. *Bacillus subtilis* NCIB 3610 encodes diverse LXG toxins to mediate CDI competition within biofilms, a process that requires the WXG100 protein YukE [35], but the role of YukE during such CDI competition is still an unexplored mystery. In comparing the inconsistent result from flow cytometry and CFU counting assay, we propose that YukE might contribute to CDI by facilitating the cell–cell adhesion process. This hypothesis is also supported by an experiment performed by Leonor García-Bayona et al. They introduced two fluorescent plasmids, mVenus and Tdtomato, into competitor and recipient strains, respectively, and subsequently used flow cytometry to measure both of the fluorescent ratios after competition. They found that 12% of the cells expressed both fluorescent proteins, providing evidence that one of the mechanisms by which CdzC mediates CDI competition is through adhesion [16], thereby supporting our hypothesis that YukE might contribute to the cell–cell adhesion process during CDI.

Since the function of contact-dependent inhibition (CDI) mediated by the LXG toxin and T7SS in *Bacillus* was discovered, its ecological role has yet to be elucidated. In this study, we showed that the expression of different *lxg* genes is significantly upregulated by root exudates produced by different hosts, indicating that SQR9 modulates its toxin production in response to the rhizosphere environment, and tailors its competitive effector based on the specific microbial community (Figure 4). This adaptive response underscores the ecological relevance of LXG toxins in rhizosphere niche competition. The rhizosphere survival challenge experiments demonstrated that the T7SS-mediated secretion of LXG toxins enhances competitiveness of SQR9 in natural rhizosphere soil (Figure 6), and highlights the importance of CDI mediated by LXG toxins in overcoming native microbial competition. Rhizobacteria survival and colonization ability in the rhizosphere is crucial to the effectiveness of microbial fertilizer, which is often limited by the bacteria competition. An in-depth analysis of the root exudation compounds that are responsible for inducing T7SS and LXGs expression will support the strategy of enhancing the survival of *Bacillus* in the rhizosphere by manipulating the T7SS with fertilizer additives.

## 4. Materials and Methods

### 4.1. Experimental Materials

*Bacillus velezensis* CLA178, *Bacillus proteolytic* TZ4, *E. coli* DH5α, *Bacillus velezensis* SQR9 (China General Microbiology Culture Collection Center, CGMCC accession number 5808), and its derived mutants were cultured at 37 °C in LB medium or MSgg medium as described in reference [39]. Antibiotics were supplemented when necessary, at the following concentrations: chloramphenicol at 10 mg/L and zeocin at 20 mg/L. Detailed information regarding the strains and plasmids used in this study is provided in Supplementary Table S1.

### 4.2. Mutant Strain Construction

Mutant strains were constructed from *B. velezensis* SQR9 based on the bacteria homologous recombination. Seven single lxg encoding genes deletion mutants (Δlxg1-ΔywqJ) were generated using a marker-free knockout method based on the counter-selectable marker *pheS* [43]. All mutants were verified by PCR and sequencing. The P43::gfp encoding gene was introduced into the Δlxg4 and Δlxg4Δantilxg4 strains by using a chloramphenicol resistance cassette for selection of the positive strain. Other mutants marked by P43::gfp or a chloramphenicol resistance cassette were introduced via the pNW33n plasmid. Antibiotics used for the selection of mutants were supplemented at the following concentrations: chloramphenicol at 10 mg/L, zeocin at 20 mg/L, and spectinomycin at 100 mg/L. Detailed information on the strains and primers used for genetic manipulation is provided in Supplementary Tables S1 and Table S2, respectively.

### 4.3. Growth Curve and Biofilm Formation Assay

For growth curve analysis, strains SQR9 WT and the seven lxg gene deletion mutants were inoculated at a 1% ratio into fresh sterile LB liquid medium, followed by transferring 200 µL bacterial suspension to a 96-well sterile plate, and the optical density at 600 nm was monitored over 24 h using a BioScreen automated growth curve instrument, setting the procedure to reading OD600 every hour. The data were plotted as growth curves. Each treatment was performed in six replicates.

For biofilm formation in liquid, strains SQR9 WT and the seven lxg gene deletion mutants were inoculated at a 1% ratio into fresh sterile MSgg medium and dispensed into sterile 24-well plates. The plates were incubated statically at 37 °C for 24 h. Once biofilm formation was observed, photographs were taken every 2 h. Four replicates were included for each treatment.

### 4.4. Competition Assay

For competition in biofilm on solid LB medium, competitor and recipient strain cells were collected and diluted to OD600 of 0.1, respectively, followed by mixing in pairs with the competitor and recipient cell suspension at ratios of 1:1, and 1 µL of the mixed bacteria suspension was spotted onto solid LB medium. Droplets were dried in a sterile environment at room temperature, then the mixed cells were allowed to form biofilm during static incubation at 30 °C for 24 h. When necessary, the competitor and recipient cells ratio was adjusted to 20:1. Three replicates were included for each treatment.

For recipient strains carrying chloramphenicol (Cm) resistance cassettes, the mixed culture was serially diluted and plated onto LB agar supplemented with 10 mg/L chloramphenicol. Then, the colony counts of each treatment were calculated, and the initial inoculum colony count was subtracted to calculate the survival of recipient strains post-competition.

For competition in liquid medium, the recipient strains carrying the P43::gfp encoding gene and the competitor strains were non-fluorescence. The competitor and recipient strains were cultured in LB liquid medium until the OD600 reached 1.0, respectively. Then, they were collected and diluted to OD600 of 0.1, followed by equally mixing in pairs and inoculation into 50 mL fresh sterile LB medium at a final concentration of OD600 0.01, and incubation at 37 °C with shaking at 170 rpm for 24 h. GFP fluorescence was measured using a Tecan Infinite M200 PRO microplate reader, Tecan, Männedorf, Switzerland. Excitation and emission wavelengths were set at 488 nm and 543 nm, respectively. Each treatment was performed with three replicates. 

### 4.5. Quantitative PCR

After competition on solid LB medium, the mixed biofilm DNA was extracted by FastPure Bacteria DNA Isolation Mini Kit (Vazyme, Nanjing, China), and the DNA concentrations were determined. Copies of SQR9 and derived mutants in the DNA were quantified by using specific primers from Qiu et al. [44]. The conserved fragment of 16S rDNA copies among SQR9, TZ4, CLA178, and DH5α was quantified by using specific primers listed in Supplementary Table S2. Data were normalized of total DNA concentration. The qPCR was performed by SYBR Premix Ex Taq (Takara, San Jose, CA, USA) with QuantStudio 6 Flex (Applied Biosystems, Waltham, MA, USA). The following PCR program was used: cDNA was denatured for 30 s at 95 °C, followed by 40 cycles of 5 s at 95 °C and 34 s at 60 °C.

### 4.6. Flow Cytometry Analysis

Each recipient strain constitutively expressed GFP fluorescence due to carrying the P43::gfp encoding gene. The post-competition ratio and after-competition ratio of recipient strains were assessed by determining the ratio of GFP-positive cells to total cells using a BD LSR Fortessa X-20 flow cytometer, Becton, Dickinson and Company, Franklin Lakes, NJ, USA. The instrument was calibrated using a negative control sample: forward scatter (FSC), side scatter (SSC), and fluorescence signal voltages were adjusted. A GFP-negative control sample was used to set the threshold (Gate), ensuring negative signals remained below 1000 and positive signals exceeded 1000. Experimental samples were analyzed at a rate of 2000 cells per second, with a total of 50,000 cells collected. Data were processed using FlowJo 10.8.1, and single-parameter histograms and dual-parameter dot plots were generated to distinguish GFP-positive and negative cell populations. The percentage of GFP-positive cells relative to the total cell count was calculated. Each treatment was performed with three replicates.

### 4.7. Rhizosphere Survival Challenge 

All tested strains carried a zeocin resistance cassette. Bacteria cells were inoculated into 5 g of fresh or sterilized rhizosphere soil at a final concentration of 10^7^ cells/g soil. Soil sterilization was performed at 121 °C for 1 h to ensure complete elimination of spores [45]. Soil samples were incubated in a light incubator under *Arabidopsis* cultivation conditions: 16 h light and 8 h dark cycles at 22 °C. After 24 h, sterile PBS was added into each soil tube, followed by vortexing at maximum speed for 10 min to harvest the bacterial suspension. Then, the suspensions were diluted to 10^−1^–10^−5^ and spread onto LB agar plates containing 20 mg/L zeocin and incubated at 37 °C for 10 h. The colony cells were calculated and normalized by soil weight to quantify the survival of each strain post-rhizosphere competition.

### 4.8. Statistical Analysis

The correlation analysis of gene transcription levels was as described by Liu et al. [36]. The collected 72 RNA-seq FPKM datasets of *B. velezensis* SQR9 from five independent experiments were normalized by SUM and transformed to relative LOG expression values. Then, the Pearson correlation coefficients between gene pairs were calculated by R (v.3.6), and a heatmap was generated. Unpaired two-tailed Student’s *t*-test analysis and data plotting were performed using GraphPad PRISM (v.8.0.1).

## 5. Conclusions

In this study, we revealed that *B. velezensis* SQR9 secreted LXG toxins to mediate CDI competition in a T7SS- and YukE-dependent manner, enhancing the rhizosphere competitiveness and providing rhizosphere adaptability.

## Data Availability

The original contributions presented in this study are included in the article/Supplementary Material. Further inquiries can be directed to the corresponding author.

## Supplementary Materials

The supporting information can be downloaded at: https://www.mdpi.com/article/10.3390/ijms26062592/s1.
