# Supplementary figures and images for: LXG Toxins of Bacillus Velezensis Mediate Contact-Dependent Inhibition in a T7SS-Dependent Manner to Enhance Rhizosphere Adaptability
Source: Int J Mol Sci. 2025 Mar 13;26(6):2592. doi: 10.3390/ijms26062592 (PMC11942605; doi:10.3390/ijms26062592)

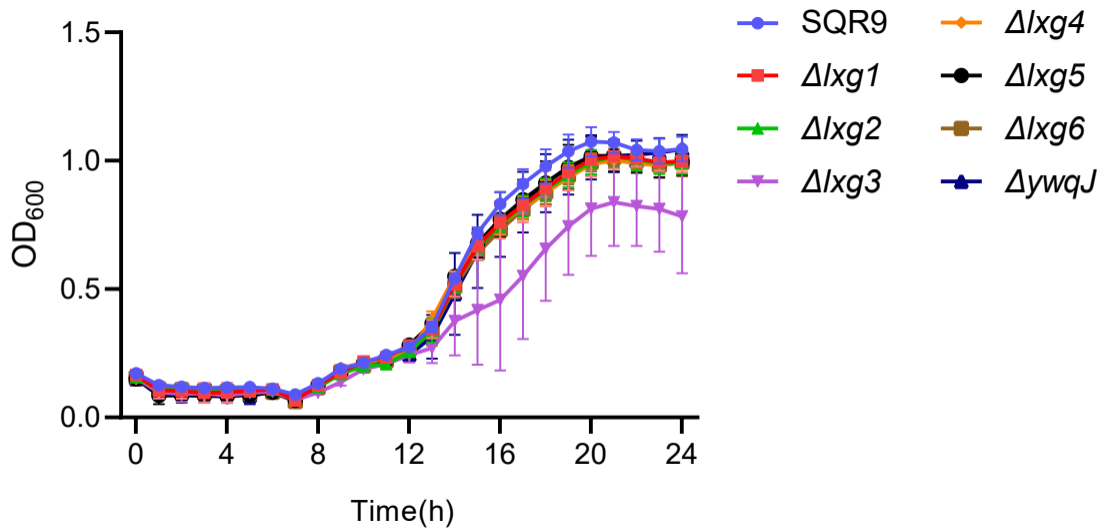

Supplement: Supplementary file 1 [file ijms-26-02592-s001.zip › Figure S1.pdf]

16 hpi

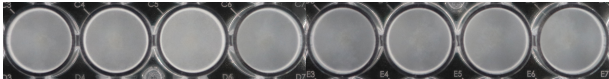

24 hpi

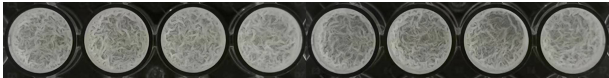

WT

$\Delta$ lxg1

$\Delta$ lxg2

$\Delta$ lxg3

$\Delta$ lxg4

$\Delta$ lxg5

$\Delta$ lxg6

$\Delta$ ywqJ

Supplement: Supplementary file 1 [file ijms-26-02592-s001.zip › Figure S2.pdf]

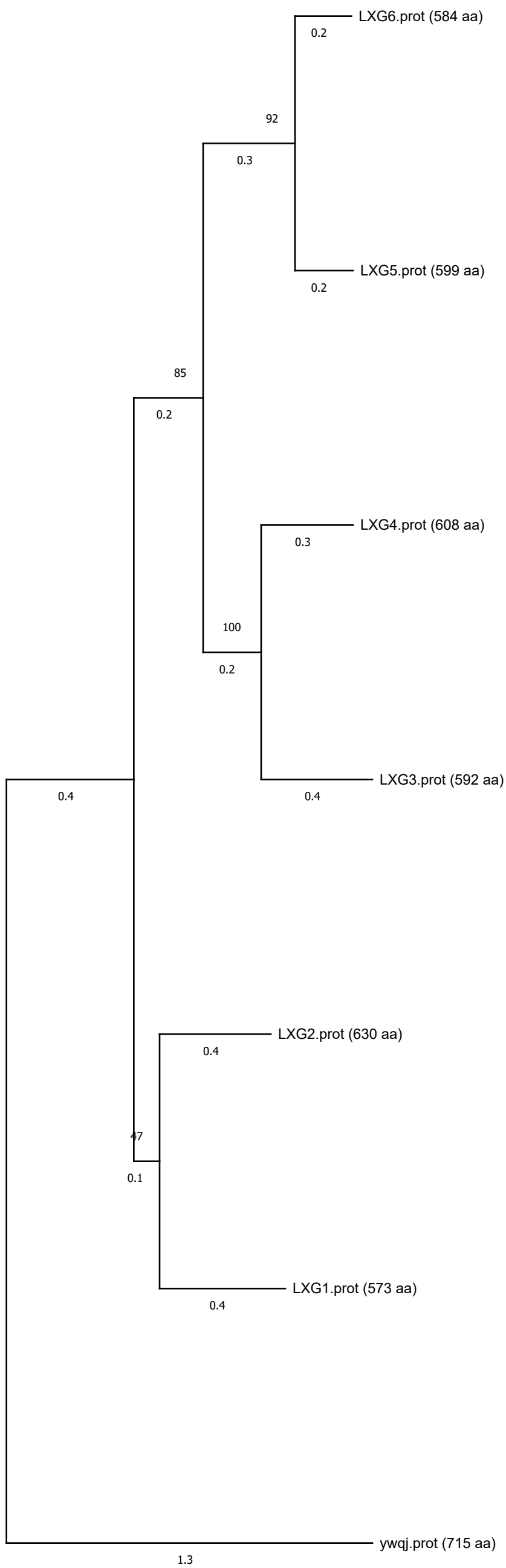

0.20

Supplement: Supplementary file 1 [file ijms-26-02592-s001.zip › Figure S3.pdf]

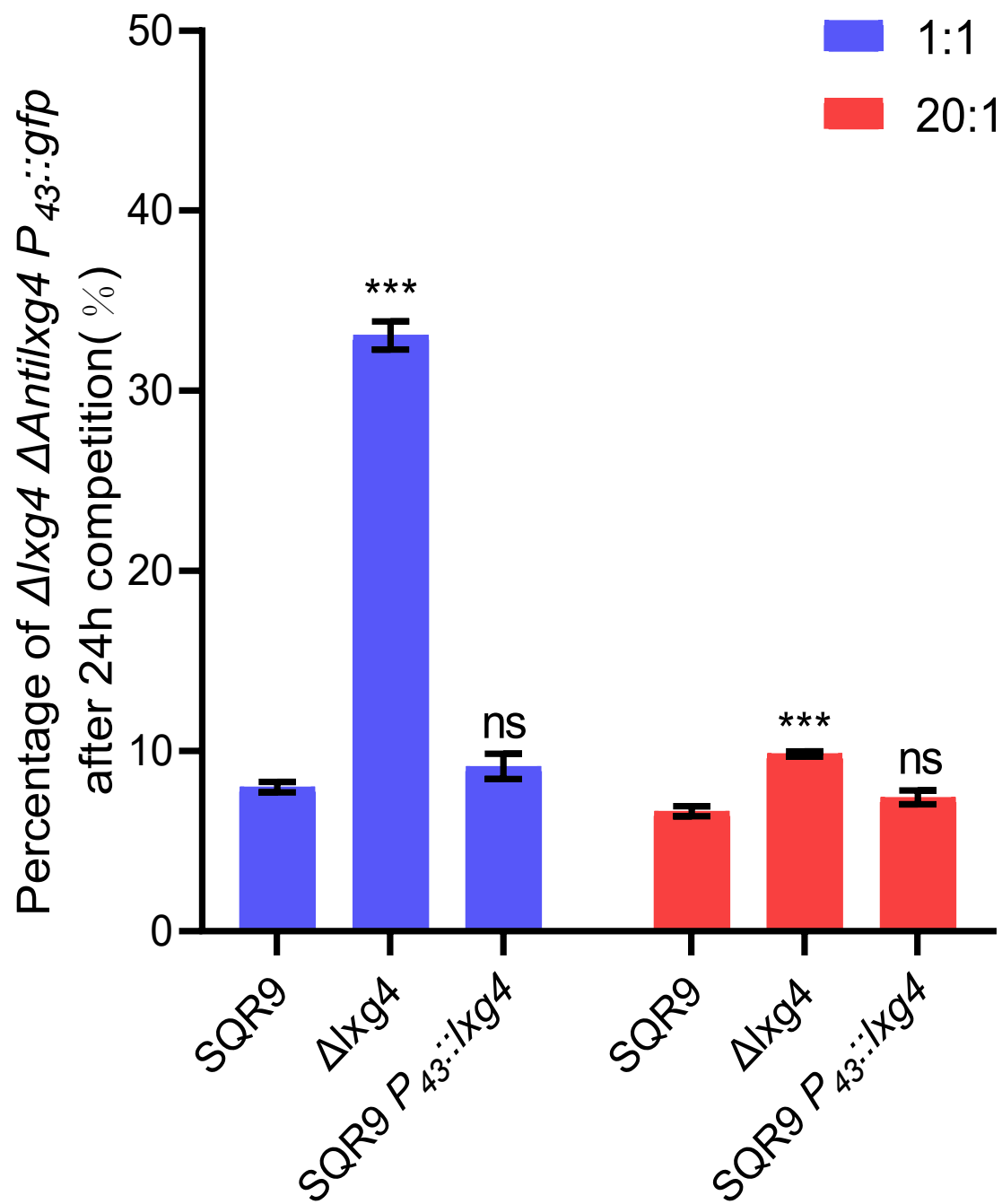

Supplement: Supplementary file 1 [file ijms-26-02592-s001.zip › Figure S4.pdf]
